# Association between nonalcoholic steatohepatitis and high serum ferritin levels in type 2 diabetes mellitus

**DOI:** 10.1590/1806-9282.20231405

**Published:** 2024-07-19

**Authors:** Tong Wang, Le He, Shaoxin Wang, Dequan Ma

**Affiliations:** 1 Tianjin Huanghe Hospital, Health Management Center – Tianjin, China.; 2 Tianjin Chest Hospital, Department of Cardiology – Tianjin, China.; 3 Tianjin Medical University, Chu Hsien-I Memorial Hospital, Tianjin Institute of Endocrinology, Department of Geriatrics – Tianjin, China.

**Keywords:** Ferritin, Nonalcoholic fatty liver disease, Fibrosis, Male, Steatosis

## Abstract

**OBJECTIVE:**

The aim of this study was to assess the role of elevated serum ferritin levels in the onset, pathological progression and prognosis of nonalcoholic fatty liver disease. Nonalcoholic fatty liver disease has been rapidly increasing worldwide. Despite extensive research on the pathogenesis of nonalcoholic fatty liver disease, a lack of sufficient clinical research on the relationship between nonalcoholic fatty liver disease and serum ferritin levels remains.

**METHODS:**

We analysed 968 patients with type 2 diabetes mellitus who underwent liver ultrasound examination and had their serum ferritin levels measured. The presence of nonalcoholic fatty liver disease and advanced liver fibrosis was determined through abdominal ultrasound examination and the nonalcoholic fatty liver disease fibrosis score.

**RESULTS:**

Compared to that in the non-nonalcoholic fatty liver disease group, the presence of hyperferritinemia was significantly more common in the nonalcoholic fatty liver disease group (83.3 vs. 56.3%, p=0.005). When patients with nonalcoholic fatty liver disease were stratified by the nonalcoholic fatty liver disease fibrosis score, those with advanced liver fibrosis exhibited a higher prevalence of hyperferritinemia (56.3, 78.9, and 88.9% for none, simple steatosis, and advanced fibrosis, respectively; p for trend=0.002). In multivariate logistic regression, liver fibrosis was independently associated with hyperferritinemia (odds ratio [OR] 1.45; 95% confidence interval [CI] 1.18–2.02; p=0.014), and this association remained significant in male patients after adjusting for other risk factors (OR 2.66; 95% CI 1.43–5.48; p=0.026).

**CONCLUSION:**

Identifying nonalcoholic fatty liver disease patients at a risk of developing nonalcoholic steatohepatitis and advanced fibrosis is crucial for implementing timely interventions and improving patient outcomes. This study highlights the potential utility of serum ferritin levels as a serum biomarker for identifying nonalcoholic steatohepatitis patients and those at a risk of late-stage fibrosis, particularly in male patients with nonalcoholic fatty liver disease.

## INTRODUCTION

Nonalcoholic fatty liver disease (NAFLD) is the most common chronic liver disease, affecting approximately 100 million people worldwide^1,2^. The spectrum of the disease ranges from hepatic steatosis to nonalcoholic steatohepatitis (NASH), which can progress to advanced liver fibrosis, cirrhosis, or hepatocellular carcinoma^3^. In addition, a significant proportion of NAFLD patients have metabolic comorbidities, which significantly increase the risk of cardiovascular disease and extrahepatic malignancies^4,5^. Notably, the prevalence of NAFLD in Asian countries is similar to and, in some cases, higher than that in Western countries, approaching 30%^6^. By 2030, the total number of NAFLD cases in China is expected to increase to 1.3158 billion, making it the country with the highest growth rate in NAFLD incidence in the world^7^.

Ferritin is a major intracellular iron storage protein^8^. The maintenance of iron homeostasis in the body is achieved through the regulation of iron ion transport and storage. Studies suggest that ferritin can enter the circulation via the classical endoplasmic reticulum/Golgi-dependent secretion pathway in hepatocytes^9^. In addition, another possible mechanism of ferritin secretion involves leakage from damaged cells^10,11^. Ferroptosis is a recently discovered iron-dependent form of cell death with distinct cellular morphology and biological mechanisms^12^. Therefore, the disruption of iron metabolism induced by ferroptosis may explain the strong association between serum ferritin (SF) and markers of hepatocellular damage. However, the relationship between SF and liver disease remains controversial. Previous reports have suggested that higher levels of SF are associated with a higher incidence of NAFLD, severe steatosis, or advanced fibrosis in patients with NAFLD^13^. Ferritin has also been shown to be a significant factor in the progression of hepatitis C to advanced liver fibrosis or even cirrhosis^14,15^. Conversely, others argue that SF levels are not very accurate in diagnosing liver fibrosis caused by NAFLD^16^. In addition, the prevalence and incidence of NAFLD are higher in China than in Western countries, although the overweight and obesity rates in the Chinese population are much lower^17^. Therefore, there is an urgent need to identify serum biomarkers for the rapid and accurate detection of NAFLD^18^.

The aim of this clinical study was to assess the role of elevated SF levels in the onset, pathological progression and prognosis of NAFLD. In addition, we would perform further stratification to investigate other potential factors that may influence SF levels to gain a deeper understanding of the relationship between NAFLD and elevated SF levels.

## METHODS

### Study population

We included a total of 1872 type 2 diabetes mellitus (T2DM) patients aged ≥18 years who underwent liver ultrasound examination and SF level measurement at Tianjin Huanghe Hospital between 2020 and 2021. Individuals with a history of coronary artery disease or ischaemic stroke, individuals with an alcohol intake greater than 140 g/week, and individuals with a history of malignancy, chronic kidney disease, thyroid dysfunction, or hepatitis B or hepatitis C infection were excluded (n=904). The analysis was conducted on the remaining 968 participants. This study was performed according to the Declaration of Helsinki and with permission from the local ethics committee (No. 2021KY-032-01).

### Anthropometric and biochemical measurements

Data on demographic characteristics, medical history, and social habits, including alcohol consumption, were obtained through self-report questionnaires at the first visit. Obesity was defined based on the Asia-Pacific criteria (BMI ≥ 25 kg/m^2^). Waist circumference was measured at the midpoint between the lower rib margin and the iliac crest during normal expiration. Blood pressure was measured using a mercury sphygmomanometer after resting for at least 5 min. Hypertension was defined as a blood pressure ≥ 140/90 mmHg or the use of antihypertensive medications. Plasma glucose concentrations were measured using the Beckman Glucose Analyser II (Beckman Instruments, Fullerton, California, USA). HbA1c was quantified using high-performance liquid chromatography (Variant II; Bio-Rad, Hercules, California, USA).

### Abdominal ultrasound examination

Abdominal ultrasound was performed by a radiologist who was blinded to laboratory and clinical data using a high-resolution ultrasound system (VISION 900; HI, Tokyo, Japan). NAFLD was defined as the presence of hepatic steatosis on ultrasound examination. In NAFLD patients, the presence of advanced liver fibrosis was determined using the NAFLD Fibrosis Score (NFS), which was calculated as follows: −1.675+0.037 × age+0.094 × BMI+1.13 × (impaired fasting glucose or diabetes)+0.99 × AST/ALT - 0.013 × platelet count - 0.66 × albumin ≥ −1.445. Simple steatosis was defined as the detection of hepatic steatosis on ultrasound examination without fibrosis predicted by the NFS.

### Serum ferritin and transferrin saturation

Serum ferritin levels were measured using an immunoradiometric assay.

### Statistical analysis

Normally distributed continuous variables are reported as the mean±standard deviation. Categorical variables are reported as frequencies. Between-group comparisons for continuous variables were performed using Student's t-test or analysis of variance (ANOVA), while the chi-square test was used for categorical variables. The odds ratio (OR) and 95% confidence interval (CI) were calculated using multivariate logistic regression. A p-value<0.05 was considered statistically significant. All statistical analyses were performed using Stata Version 17.0.

## RESULTS

### Clinical and serum ferritin levels

Clinical characteristics and SF levels were stratified according to the presence of NAFLD, as summarized in Table 1. The mean age was 55.8±10.2 years, and 654 participants (67.56%) were male. The mean duration of T2DM was 7.8±5.6 years. NAFLD was present in 533 participants (55.1%). Compared to those in the non-NAFLD group, participants in the NAFLD group had significantly higher BMIs and waist circumferences and were more likely to have metabolic syndrome (all p<0.001). Fasting glucose and HbA1c levels were higher in patients in the NAFLD group (p<0.001). Serum cholesterol, triglyceride, and liver enzyme (AST and ALT) levels were significantly elevated in the NAFLD group (all p<0.001). The NAFLD group had higher SF levels than the non-NAFLD group (p<0.001). Thus, 689 participants (71.2%) had elevated SF levels, and the prevalence was significantly higher in the NAFLD group than in the non-NAFLD group (83.3 vs. 56.3%, p=0.005).

**Table 1:** Clinical and echocardiographic parameters.

		Non-NAFLD	NAFLD	p-value
		(n=435)	(n=533)	
Age (years)		55.60±13.66	56.01±12.86	0.3483
Gender (%)				
	Male	65.75	69.04	0.128
	Female	34.25	30.96	
Age (years)				
	≥50	59.78±8.27	59.04±7.38	0.0779
	<50	37.13±8.06	37.70±7.67	0.0831
	Duration of diabetes mellitus (years)	7.60±5.12	8.67±3.21	0.6827
	Systolic BP (mmHg)	125.26±19.02	132.57±17.85	<0.0001
	Diastolic BP (mmHg)	78.81±19.64	83.03±9.82	<0.0001
	BMI (kg/m^2^)	24.09±3.53	27.92±3.42	<0.0001
	Waist circumference (cm)	88.00±10.17	99.80±8.07	<0.0001
Laboratory features				
	Total cholesterol (mmol/L)	5.05±0.98	5.89±0.99	<0.0001
	HDL-cholesterol (mmol/L)	1.40±0.28	1.28±0.25	<0.0001
	LDL-cholesterol (mmol/L)	3.01±0.77	3.05±0.76	0.0988
	Triglyceride (mmol/L)	1.61±1.04	2.15±1.18	<0.0001
	Glucose (mmol/L)	5.12±1.17	5.82±1.91	<0.0001
	HbA1c (%)	5.37±0.87	5.94±1.01	<0.0001
	ALT (IU/L)	24.64±9.88	28.02±11.67	<0.0001
	AST (IU/L)	28.95±21.42	41.79±30.68	<0.0001
	γGT (IU/L)	27.70±26.06	36.89±26.89	<0.0001
	Serum albumin (g/L)	46.92±2.21	46.61±2.11	<0.0001
	Platelet count (10^9^/L)	234.52±55.91	229.34±49.76	0.0341
	Serum creatinine (μmol/L)	72.15±15.68	76.09±17.17	<0.0001
	serum urea nitrogen	5.15±1.33	5.35±1.30	<0.0001
	Uric acid (μmol/L)	338.85±79.43	381.70±80.05	<0.0001
	Ferritin (ng/mL)	124.38±152.01	325.90±157.03	<0.0001

Values are presented as mean±standard deviation or number (%). NAFLD: nonalcoholic fatty liver disease; BMI: body mass index; BP: blood pressure; HbA1c: glycosylated hemoglobin; HDL-C: high density lipoprotein cholesterol; LDL-C: low density lipoprotein cholesterol; ALT: alanine aminotransferase; AST: aspartate transaminase; γGT: gamma glutamyl transpeptidase.

### Association between type 2 diabetes mellitus-associated hepatic steatosis and elevated serum ferritin levels

The prevalence of elevated SF levels increased gradually with the severity of hepatic steatosis (56.3, 76.0, and 88.2% for none, mild, and moderate/severe, respectively; p for trend=0.005). Among individuals stratified by the NFS, participants with advanced liver fibrosis had a significantly higher prevalence of elevated SF levels than participants without steatosis (56.3, 78.9, and 88.9% for none, simple steatosis, and advanced fibrosis, respectively; p<0.001 for advanced fibrosis vs. none, p for trend=0.002).

**Figure 1:**
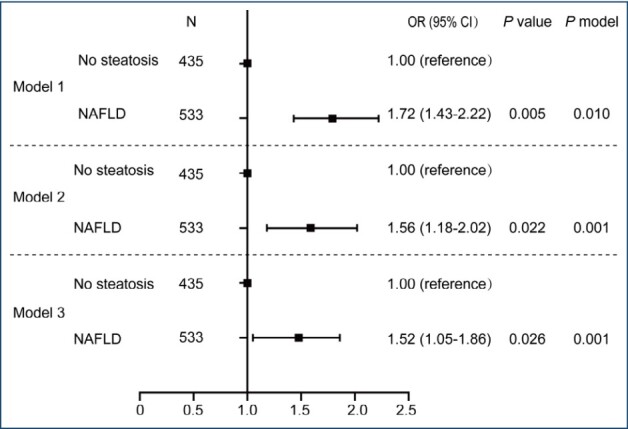
Adjusted odds ratio for hyperferritinemia by the presence of nonalcoholic fatty liver disease. Multivariable logistic regression in all subjects. Model 1, unadjusted; model 2, adjusted for age, BMI, hypertension, diabetes mellitus duration, fasting glucose, triglyceride, and cholesterol; model 3, further adjusted for sex. OR: odds ratio; CI: confidence interval; NAFLD: nonalcoholic fatty liver disease; BMI: body mass index.

To assess whether NAFLD is independently associated with elevated SF levels in T2DM patients, multivariable logistic regression analysis was performed. Compared to the absence of NAFLD, the presence of NAFLD was positively associated with a higher likelihood of elevated SF levels (OR, 1.56; CI, 1.18–2.02; p=0.022) after adjustment (Figure 1, Model 2). This association remained significant but was attenuated when the model was further adjusted for sex (OR 1.52; 95%CI 1.05–1.86; p=0.026) (Figure 1, Model 3). In the subgroup analysis, NAFLD did not have a heterogeneous effect on hyperferritinemia depending on the HbA1c level, duration of diabetes, hypertension, BMI, age, or sex (p>0.05 for all interaction terms with NAFLD). Notably, this association was significant in male patients (OR 2.58; 95%CI 1.24–5.48; p=0.021), whereas it was not significant in the female population (p=0.585, interaction term p=0.032).

### Relationship between advanced liver fibrosis and elevated serum ferritin levels in type 2 diabetes mellitus patients

**Figure 2:**
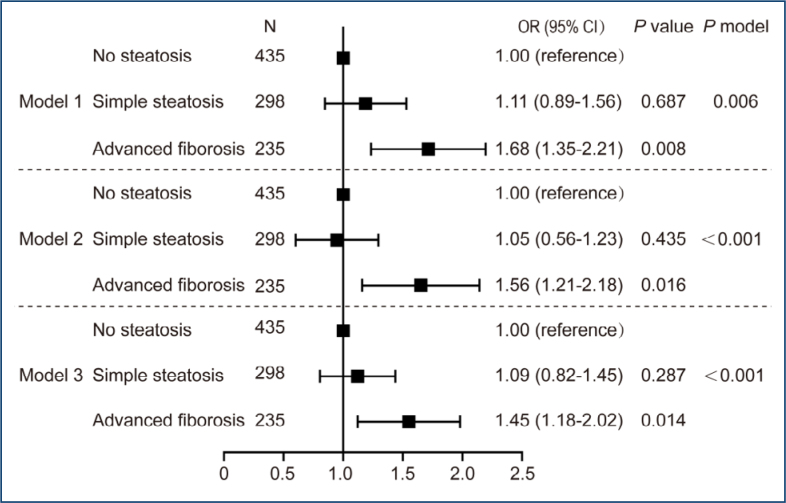
Adjusted odds ratio for hyperferritinemia by the presence of advanced liver fibrosis with nonalcoholic fatty liver disease. Multivariable logistic regression in all subjects. Model 1, unadjusted; model 2, adjusted for age, BMI, hypertension, diabetes mellitus duration, fasting glucose, triglyceride and cholesterol; model 3, further adjusted for sex. OR: odds ratio; CI: confidence interval; NAFLD: nonalcoholic fatty liver disease; BMI: body mass index.

As shown in Figure 2, we investigated the relationship between the presence of advanced liver fibrosis with NAFLD and hyperferritinemia. In the stratification of NAFLD patients using the NFS score, advanced liver fibrosis was associated with hyperferritinemia (OR, 1.45; CI, 1.18–2.02; p=0.014) after adjustment (Figure 2, Model 3). No significant heterogeneity was found among the various subgroups defined by glycaemic control, duration of diabetes, hypertension, BMI, sex, and age (p>0.05 for all interaction terms with the NFS). However, this association disappeared in female patients (p=0.447), while it was maintained in male patients (OR 2.66; 95%CI 1.43–5.48; p=0.026, interaction term p=0.019).

## DISCUSSION

First, we observed significantly higher SF levels in NAFLD patients than in non-NAFLD patients. This is consistent with previous research findings^13^, suggesting the presence of disrupted iron metabolism in the development of NAFLD. Elevated SF levels may serve as predictive markers of NAFLD development, reflecting iron accumulation and abnormal iron distribution in the body. Furthermore, we found that NAFLD patients with advanced liver fibrosis were more likely to exhibit abnormal SF levels than patients with only steatosis, and the prevalence of elevated SF levels increased gradually with the severity of NAFLD. This further supports the relationship between elevated SF levels and NAFLD severity and liver fibrosis progression. Therefore, our study confirms the value of SF as a noninvasive biomarker for identifying NAFLD patients. Impaired or enhanced ferritin function can lead to the abnormal accumulation of iron ions, increasing the risk of ferroptosis. Elevated SF levels or disturbances in iron metabolism may increase the risk of cell ferroptosis^19-23^.

Subsequently, we further conducted multivariate regression analysis in our study, which indicated that the association between NAFLD and elevated SF levels persisted after adjusting for other potential confounding factors. This further supports the independent relationship between NAFLD and elevated SF levels. This association may be influenced by sex, as the association between NAFLD and elevated SF levels was more significant in male patients, while no such association was observed in female patients in the subgroup analysis based on sex. Liang et al.^24^ found that ferroptosis is modulated by the differential effects of sex hormones. The study identified MBOAT1 (an oestrogen receptor) and MBOAT2 (an androgen receptor) as novel regulators of ferroptosis by reshaping phospholipids. Compared to males, females exhibit significant resistance to ferroptosis in vivo; this resistance is significantly influenced by the female sex hormone environment. Based on in vivo single-cell resolution and unbiased computational inference, Shintaro et al.^25^ found that the resilience of females to ferroptosis is both constitutive and adaptive. It is speculated that the regulation of ferroptosis sensitivity involves multiple interactions between cells and their environment. The promotion of BCL6 increased the susceptibility of male mice to NAFLD, suggesting that males limit their immunopathology^26^. The circadian rhythm can protect the liver by influencing hormone levels, and its disruption can lead to the liver undergoing "gender switching"^27^.

Our study has several advantages. This study explored the relationship between the severity of liver steatosis and/or advanced liver fibrosis and high ferritin levels in T2DM patients, which can lead to the study of new ferroptosis pathogenic mechanisms and expand our understanding of the pathogenesis of NAFLD. Compared to those of previous studies, the large sample size of our study allowed us to further investigate the impact of liver fibrosis in subgroup analysis, and we found that males are more prone to having high ferritin levels, while females are less susceptible, possibly due to ferroptosis surveillance being differentially regulated by sex hormones. However, we also acknowledge some limitations in our study. First, the cross-sectional design precludes causal inferences between NAFLD and high ferritin levels. Second, due to its invasive nature, liver biopsy to confirm the presence of steatohepatitis was not feasible. Instead, we used the NFS, which has been well validated and widely used for screening for advanced liver fibrosis in NAFLD patients. Finally, this was a single-centre study from a Chinese hospital, and caution should be exercised when extrapolating the results to different clinical settings.
